# Regulatory T Cells in Peripheral Blood and Cerebrospinal Fluid of Syphilis Patients with and without Neurological Involvement

**DOI:** 10.1371/journal.pntd.0002528

**Published:** 2013-11-07

**Authors:** Kang Li, Cuini Wang, Haikong Lu, Xin Gu, Zhifang Guan, Pingyu Zhou

**Affiliations:** STD Institute, Shanghai Skin Disease Hospital, Shanghai, People's Republic of China; University of Washington, United States of America

## Abstract

**Background:**

Syphilis, a sexually transmitted disease caused by spirochetal bacterium *Treponema pallidum*, can progress to affect the central nervous system, causing neurosyphilis. Accumulating evidence suggest that regulatory T cells (Tregs) may play an important role in the pathogenesis of syphilis. However, little is known about Treg response in neurosyphilis.

**Methodology/Principal Findings:**

We analyzed Treg frequencies and Transforming Growth Factor-β (TGF-β) levels in the blood and CSF of 431 syphilis patients without neurological involvement, 100 neurosyphilis patients and 100 healthy donors. Suppressive function of Tregs in peripheral blood was also assessed. Among syphilis patients without neurological involvement, we found that secondary and serofast patients had increased Treg percentages, suppressive function and TGF-β levels in peripheral blood compared to healthy donors. Serum Rapid Plasma Reagin (RPR) titers were positively correlated with Treg numbers in these patients. Compared to these syphilis patients without neurological involvement, neurosyphilis patients had higher Treg frequency in peripheral blood. In the central nervous system, neurosyphilis patients had higher numbers of leukocytes in CSF compared to syphilis patients without neurological involvement. CD4^+^ T cells were the predominant cell type in the inflammatory infiltrates in CSF of neurosyphilis patients. Interestingly, among these neurosyphilis patients, a significant decrease in CSF CD4^+^ CD25^high^ Treg percentage and number was observed in symptomatic neurosyphilis patients compared to those of asymptomatic neurosyphilis patients, which may be associated with low CSF TGF-β levels.

**Conclusions:**

Our findings suggest that Tregs might play an important role in both bacterial persistence and neurologic compromise in the pathogenesis of syphilis.

## Author Summary

Syphilis, caused by the bacterium *Treponema pallidum*, can progress to affect the central nervous system (CNS) and cause damage in the brain and spinal cord, which is called neurosyphilis. While many affected neurosyphilis patients may not have any symptoms, some of the patients will develop severe symptoms that can be life-threatening. Regulatory T cells (Tregs) are a subpopulation of CD4^+^ T cells functioning in suppression of immune-mediated bacterial clearance and tissue damage. In this study, we conduct a comparative analysis of regulatory T cells (Tregs) in the blood and cerebrospinal fluid (CSF) of syphilis patients without neurological abnormalities, and neurosyphilis patients with or without symptoms. Our results show that neurosyphilis patients had higher Treg percentage in blood than syphilis patients without neurological involvement, suggesting that neurological progression in syphilis patients is associated with an increase in blood Treg numbers. Strikingly, a decrease in Treg percentage and numbers in CSF of symptomatic neurosyphilis patients was observed compared to asymptomatic neurosyphilis patients. These results may implicate reduced CNS Treg response as a factor underlying the development of symptoms in some neurosyphilis patients. Our findings highlight a discordant Treg response in blood and CSF in symptomatic neurosyphilis patients and further underscore the fascinating complexity of immune response in syphilis.

## Introduction

China has experienced an expanding epidemic of syphilis infection in the last 10 years [1], [2]. In 2011, the national incidence rate was 32.04 per 100,000 population and 429,677 new cases were reported [3]. This sexually transmitted disease has reemerged as a significant public health issue in China due to its serious, irreversible sequelae [4] and its strong association with HIV infection [5]. The rapid rise in syphilis rates in China highlight the importance of understanding of the pathogenesis of syphilis and its complications.

The spirochetal bacterium, *Treponema pallidum (T. pallidum)*, is the etiologic agent of syphilis [6], [7]. After *T. pallidum* infection, mammalian hosts mount robust humoral and cellular immune responses aimed at spirochetal clearance [8], [9], [10], [11]. However, *T. pallidum* has the ability to escape the host immune response and establish persistent infection. There are several strategies used by the spirochete to resist host immune effector mechanisms including poor antigenicity [12], [13], antigenic variation of membrane proteins [14], [15], [16], and impaired antibody-mediated opsonization [17]. Interestingly, several studies have demonstrated that *T. pallidum* may also actively harness host immune suppression mechanisms to facilitate persistence and dissemination [18], [19]. A recent study has demonstrated that *T. pallidum* antigen TpF1 could promote development of regulatory T cells (Tregs) in the patients with secondary syphilis [18]. Tregs represent a unique population of CD4^+^ T cells with potent immune suppressive activity [20], [21]. This regulatory CD4^+^ T cell population is classically defined by high expression of CD25 (IL-2 receptor α-chain) [22]. The forkhead family transcription factor Foxp3, the most definitive signature, is critical for Treg development and function [23]. Emerging evidence from human patients and animal models has demonstrated that Tregs contribute to impaired immune responses and chronic infection with diverse organisms [24], including mycobacterium tuberculosis [25], helicobacter pylori [26], hepatitis B virus [27], [28], HIV [29], and plasmodium falciparum [30]. The enhanced Treg response in early syphilis patients may down-regulate immune effector function to allow survival of *T. pallidum* within the host.

*T. pallidum* infection can infect many organs, including central nervous system (CNS). This form of syphilis is termed neurosyphilis. Neurosyphilis may affect the meninges or brain or spinal cord parenchyma and may be asymptomatic or symptomatic [4], [31]. Meningeal neurosyphilis usually appears during the first few years of *T. pallidum* infection. Patients with meningeal neurosyphilis may be manifested by meningitis (headache, stiff neck, and cranial nerve abnormalities) or meningovasculitis (focal CNS ischemia or stroke). Parenchymal neurosyphilis, presenting as general paresis and tabes dorsalis, occur in the later course of the disease, often decades after the primary infection [4], [32]. The mechanisms underlying the development of symptomatic neurosyphilis in some patients are largely unknown.

Previous studies have extensively characterized immune cell infiltrates of early syphilis lesions [8], [9], [10] and indicated that the clinical manifestations of early syphilis result from collateral tissue damage caused by host immunity to *T. pallidum* [6], [33]. However, little is known about the immune response in neurosyphilis patients. In the present study, we performed a comparative analysis of Tregs in peripheral blood and cerebrospinal fluid (CSF) from neurosyphilis patients and syphilis patients without neurological involvement. We found that symptomatic neurosyphilis patients had lower Treg frequencies and numbers in CSF compared to asymptomatic neurosyphilis patients, indicating that an immunopathological mechanism might be present in the onset of neurological symptoms.

## Methods

### Ethics Statement and Subjects

This study was performed at the Shanghai Skin Disease Hospital between June 2009 and Jan 2012. The hospital is located in central Shanghai, where the syphilis prevalence is highest in China [1]. The Sexually Transmitted Diseases (STD) center in this hospital is the major STD clinic in Shanghai, which provides screening, diagnosis and treatment for most sexually transmitted diseases, including syphilis. As one of the biggest STD centers in China, more than 300 patients are served in this clinic per day. Although most of our patients are walk-in, some are referred to our clinic by their doctors at other hospitals across the country. This study was approved by the Ethics Committee of the Shanghai Skin Disease Hospital. Written informed consent was obtained from all participants.

Syphilis was determined based on medical history, physical, neurological and psychiatric symptoms and signs, and the results of nontreponemal and treponemal serological tests. The excluded criteria include HIV; prior syphilis or syphilis treatment (except in the serofast syphilis group); history of systemic inflammatory, autoimmune disease, other underlying acute or chronic disease, were receiving anti-inflammatory medications, were immunocompromised, or use of antibiotics or immunosuppressive medications in the last four weeks. Peripheral blood was collected from all healthy donors and syphilis patients. Lumbar punctures were encouraged to be performed if i) patients had neurological or psychiatric signs or symptoms, ii) patients whose serum RPR≥1∶32, regardless of stage or presentation, iii) patients whose serofast state was more than 2 years and who are anxious regarding their serofast state. 100 healthy donors, who visited Shanghai Skin Disease Hospital voluntarily for STD prevention and a medical check-up, were recruited to the study. All healthy control subjects were negative for HIV and serological tests for syphilis.

### Diagnostic Criteria for Primary, Secondary, Latent and Serofast Syphilis

*Primary syphilis*: i) Chancres or ulcers; and/or ii) detection of spirochetes in a dark-field microscopy examination; and iii) positive RPR confirmed by *Treponema pallidum* particle agglutination assay (TPPA); and iv) absence of other causes of genital ulcers, including herpes simplex virus (HSV) infections. *Secondary syphilis*: i) positive RPR confirmed by TPPA; and ii) skin or mucocutaneous lesions; *Latent syphilis*: i) positive RPR confirmed by TPPA; and ii) without skin or mucocutaneous lesions or any symptoms of syphilis; *Serofast syphilis*: i) previously treated syphilis of any stage; ii) an appropriate 4-fold decline in serum RPR titer at 6 months after treatment (Benzathine penicillin 2.4 MU/qw im for 2 or 3 weeks or procaine penicillin 0.8 MU/day im for 15 days in most cases, if patient allergic to penicillin ceftriaxone 250 mg/day im for 10 days would be as an alternative); iii) persistently reactive serum RPR two or more years after treatment; iv) no evidence of reinfection. The clinical and laboratory characteristics of 71 patients with primary syphilis, 136 patients with secondary syphilis, 127 patients with latent syphilis, and 97 patients with serofast syphilis were shown in Table 1.

**Table 1:** Clinical and laboratory characteristics of 100 healthy donors and 431 syphilis patients.

	Healthy controls	Primary syphilis	Secondary syphilis	Latent syphilis^a^	Serofast syphilis
**No. of Cases**	100	71	136	127	97
**Male No. (%)**	70 (70)	64 (90.1)	70 (51.5)	54 (42.5)	34 (35.1)
**Median Age (IQR)**	32 (28–43)	47 (34–52)	33 (27–46)	35 (28–51)	38 (29–55.5)
**Duration^b^ (IQR)**	—	14 (10–30)^c^	30 (14–39)^c^	1 (0.5–5)^d^	24 (12–36)^d^
**Median Serum RPR titer (IQR)**	ND	16 (4–32)	64 (64–128)	16 (8–32)	4 (4–8)
**Median CSF protein, mg/dL (IQR)**	ND	30 (25–38)	24 (19–31)	25 (18–31)	25 (18.3–33)
**CSF VDRL Positive (%)**	ND	0 (0)	10 (7.4)	10 (7.9)	1 (1.0)
**CSF VDRL negative CSF-TPPA positive (%)**	ND	0 (0)	6 (4.4)	8 (6.3)	15 (15.5)

Data are no. (%) of patients, unless otherwise indicated.

a includes 127 subjects with early latent syphilis. The cut-off for early and late latent syphilis is two years.

b Duration of clinical manifestations;

c ,days;

d ,months.

Abbreviations: IQR, interquartile range; RPR, rapid plasma reagin; VDRL, venereal disease research laboratory; TPPA, *Treponema pallidum* particle agglutination assay; ND, Not Done.

### Diagnostic Criteria for Neurosyphilis

All neurosyphilis patients have positive serum RPR and TPPA tests. The diagnosis of c*onfirmed neurosyphilis* also includes reactive CSF-VDRL (Venereal Disease Research Laboratory) and CSF-TPPA tests in the absence of substantial contamination of CSF with blood. Presumptive neurosyphilis was defined as nonreactive CSF-VDRL but reactive CSF-TPPA with either or both of the following: i) CSF protein concentration >45 mg/dL or CSF white blood cell (WBC) count ≥8/µL in the absence of other known causes for these abnormalities; ii) neurological or psychiatric manifestations consistent with neurosyphilis without other known causes for these abnormalities. Fourteen patients with presumptive neurosyphilis were also included in the study and the data of these patients were combined with those of confirmed neurosyphilis patients for analysis. In the case of presumptive neurosyphilis, the patient has a nonreactive CSF-VDRL test plus a reactive CSF-TPPA along with either or both of the following: (i) elevated CSF proteins (normal: 15–45 mg/dL) or elevated CSF white blood cell (WBC) count (normal: <8/µL) in the absence of other known causes of the abnormalities; (ii) clinical neurological or psychiatric manifestations without other known causes of these clinical abnormalities.

Neurosyphilis is categorized as asymptomatic, meningeal (meningitis and meningovasculitis) and parenchymal (general paresis and tabes dorsalis). *Asymptomatic neurosyphilis* is defined by the presence of CSF abnormalities consistent with neurosyphilis and the absence of neurological and psychiatric signs or symptoms. *Meningitis* is diagnosed by CSF abnormalities and headache, stiff neck, nausea, or cranial neuropathies. *Meningovasculitis* is defined by clinical features of meningitis and stoke with or without neuroradiological confirmation. *General paresis* is characterized by personality changes, dementia and psychiatric symptoms including mania or psychosis. *Tabes dorsalis* is characterized by sensory loss, ataxia, lancinating pains, and bowel and bladder dysfunction. All patients diagnosed with neurosyphilis should have no other known causes for these clinical abnormalities. The features of 100 neurosyphilis patients are shown in Table 2. These patients are mutually exclusive of those in Table 1.

**Table 2:** Clinical and laboratory features for 100 neurosyphilis patients.

Type of Neurosyphilis	Asymptomatic Neurosyphilis^a^	Meningeal neurosyphilis		Parenchymal neurosyphilis	
		Meningitis	Meningovasculitis	General paresis	Tabes dorsalis
**No. of cases**	52	8	2	34	4
**Male No. (%)**	31 (59.6)	8 (100)	2 (100)	34 (100)	3 (75)
**Median Age (IQR)**	53 (43–60)	56 (48–63)	42 (41–43)	52.5 (46–59)	57 (49–63.5)
**Duration, years (IQR)**	0.5 (0.5–1.5)	1(0.5–1.5)	1.25 (1.0–1.5)	1 (0.5–2)	4 (1–5)
**Median serum RPR titer (IQR)**	32 (16–64)	64 (16–64)	256 (256–512)	64 (8–128)	32 (32–32)
**Median CSF protein, mg/dL (IQR)**	31 (26–43)	45.5 (28–66)	30 (20–40)	57 (43.3–76.5)	39.5 (14.5–68.3)
**Median CSF WBC, cells/µL (IQR)**	6(3–13)	8(3–14)	8 (3–20)	6 (2–15)	14 (5–32)
**CSF VDRL Positive (%)**	43 (79.6)	7 (87.5)	2 (100)	32 (94.1)	3 (75)
**CSF VDRL negative CSF-TPPA positive (%)**	11 (20.4)	1 (12.5)	0 (0)	2 (5.9)	1 (25)

Data are no. (%) of patients, unless otherwise indicated.

a includes 1 subject with primary syphilis, 20 subjects with secondary syphilis, 3 subjects with early latent and 28 subjects with late latent or unknown duration.

**Abbreviations**: WBC, white blood cells.

### Flow Cytometric Analysis

Peripheral blood mononuclear cells (PBMC) were isolated from whole blood from syphilis, neurosyphilis patients and healthy donors via density centrifugation over Lymphoprep (Axis-Shield). CSF was centrifuged and stained immediately at 4°C after spinal tap. The volume was 5 mL. Multicolor fluorescence activated cell sorting (FACS) analysis was performed using the following antibodies: PE-, FITC-, PerCP, or PE-Cy5-conjugated antibodies against human CD45 (Biolegend), CD3 (Biolegend), CD4 (Biolegend), CD25 (Biolegend). For Foxp3 staining, cells were stained using One Step Staining Human Treg Flow Kit (Biolegend) according to the manufacturer's protocols. Cells were assessed with FACScalibur (Becton Dickinson) or Epics XL (Beckman Coulter) cytometers as previously described [34]. For CSF samples, acquisition of ≥5,000 events for gated CD45^+^ cells was performed. The CSF Treg number was defined as the total number of CSF cells multiplied by the percentage of Tregs identified by flow cytometry. Data were analyzed using FlowJo software (Tree Star).

### Proliferation and Suppression Assay

Treg suppression assay was performed as described [35], [36]. Briefly, PBMC were used for CD4^+^ CD25^+^ and CD4^+^ CD25^−^ T cell isolation using a Regulatory T Cell Isolation Kit according to the manufacturer's instruction (Miltenyi Biotec). Purity of the cell fractions as determined by flow cytometry was >90%. Purified CD4^+^ CD25^−^ T responder cells (5×10^4^ cells/well) were incubated in RPMI 1640 medium with 10% FBS in 96-well U-bottom plates precoated with anti-CD3 antibody (1 µg/mL; eBioscience). To assess suppressive ability, purified autologous CD4^+^ CD25^+^ T cells were added, at a CD25^+^/CD25^−^ ratio of 1∶1, 1∶2, 1∶4, or 1∶8. All cells were cultured in a final volume of 200 µl in the presence of 2×10^4^ irradiated allogeneic PBMC/well. After 4 days of culture, [^3^H] thymidine (Amersham) was added for an additional 18 h to each well. [^3^H] thymidine incorporation was measured using a liquid scintillation counter. Percent inhibition of proliferation was determined as (1- [^3^H] thymidine incorporation of CD25^+^ and CD25^−^ T cells coculture/[^3^H] thymidine incorporation of CD25^−^ T cells alone)×100.

### Measurement of Cytokine Levels

Serum and CSF TGF-β1 levels were determined using Human TGF-β1 ELISA kit from eBioscience.

### Statistical Analysis

We performed statistical analysis using GraphPad Prism version 5.01 (GraphPad Software). All datasets were first assessed for deviation from a normal distribution using the D'Agostino-Pearson omnibus normality test. Non-normally distributed variables were compared between groups using the nonparametric Kruskal–Wallis test followed by Dunn's multiple comparison tests. If the variables were approximately normally distributed, differences between experimental groups were analyzed using one-way ANOVA followed by Bonferroni test for the selected pairs. Pearson correlation analysis was used to determine the relationship between the frequency of CD4^+^ CD25^high^ Treg and other parameters. A value of P<0.05 was considered significant.

## Results

### Treg Percentages Are Elevated in the Peripheral Blood of Primary, Secondary, Latent and Serofast Syphilis Patients

**Figure 1:**
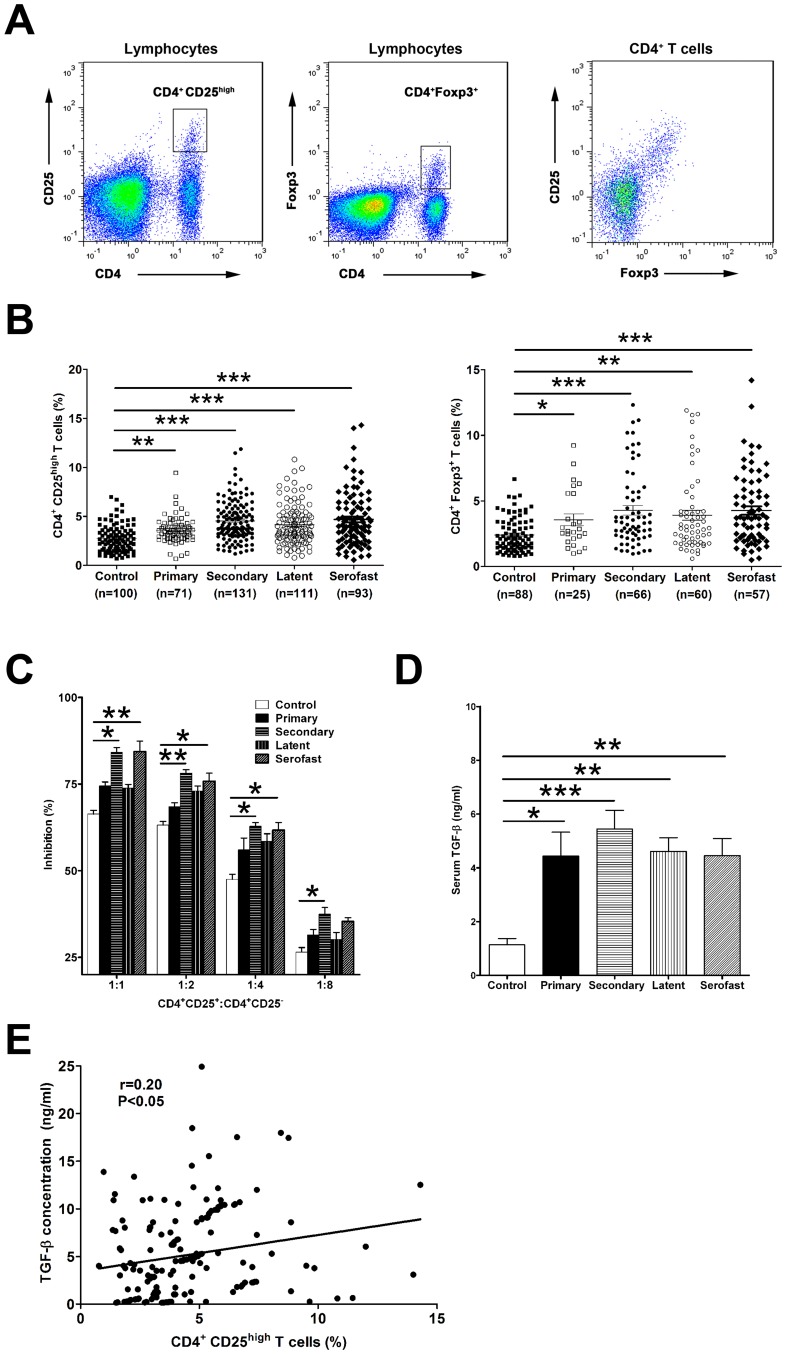
Treg activity in the peripheral blood of early and serofast syphilis patients. (**A**) Peripheral blood mononuclear cells (PBMC) were stained for flow cytometric analysis and lymphocytes were gated according to forward and side scatter characteristics. Representative plots show the gates of CD4^+^ CD25^high^ and CD4^+^ Foxp3^+^ T cells in the lymphocyte gate. CD25 and Foxp3 expression among CD4^+^ T cells are also shown. (**B**) The percentage of CD25^high^ and Foxp3^+^ among CD4^+^ T cells in peripheral blood of healthy controls, and in patients with primary, secondary, latent and serofast syphilis. Individual frequencies for every patient analyzed are shown. (**C**) CD4^+^ CD25^+^ T cells and CD4^+^ CD25^−^ T cells were purified from peripheral blood of healthy donors, and patients with primary, secondary, latent and serofast syphilis. The mean percent inhibition of the proliferative response by CD4^+^ CD25^+^ T cells derived from three to five individuals per group is shown. Results are representative of three independent experiments. (**D**) Serum concentrations of TGF-β were measured in healthy controls, and in patients with primary, secondary syphilis, latent, and serofast syphilis. Results represent the mean or the mean ± SEM. (**E**) Correlation of serum TGF-β concentration with circulating CD4^+^ CD25^high^ Treg frequencies in syphilis patients (n = 176). Each dot represents an individual patient. B, Kruskal-Wallis test; C and D, One-way ANOVA; E, Pearson's correlation. *, P<0.05; **, P<0.01; ***, P<0.001.

Human Tregs were identified as CD4^+^CD25^high^ or CD4^+^Foxp3^+^ T cells [20], [21]. The representative gating strategy for CD4^+^ CD25^high^ and CD4^+^ Foxp3^+^ T cells are depicted in Figure 1A. The majority of Foxp3^+^ T cells co-expressed high levels of CD25 (Figure 1A). The baseline frequency of CD25^high^ Tregs among CD4^+^ T cells in PBMCs from healthy individuals was 2.7%±0.1% (Figure 1B). A comparison between syphilis patients and healthy individuals revealed a 1.3-fold increase in mean frequency of CD4^+^ CD25^high^ T cells in primary syphilis patients (3.6%±0.2%, p<0.01), 1.7-fold increase in secondary syphilis patients (4.5%±0.2%, p<0.001), 1.5-fold increase in early latent syphilis patients (4.1%±0.2%, p<0.001), and 1.7-fold increase in serofast syphilis patients (4.7%±0.3%, p<0.001) (Figure 1B). Consistently with CD25 expression, the highest percentage of Foxp3^+^ Tregs among CD4^+^ T cells were observed in patients with secondary syphilis (4.3%±0.4%, p<0.001) and serofast syphilis (4.3%±0.3%, p<0.001) patients, followed by latent syphilis (3.9%±0.4%, p<0.01) and primary syphilis patients (3.6%±0.4%, p<0.05), which were all significantly higher than healthy donors (2.3%±0.1%) (Figure 1B).

### Treg Suppressive Function Is Increased in the Peripheral Blood of Secondary and Serofast Syphilis Patients

We next investigate the suppressive function of Tregs from syphilis patients on T cell proliferation. CD4^+^ CD25^+^ suppressor T cells were cocultured with autologous CD4^+^ CD25^−^ T responder cells at different ratios (suppressor/responder ratios: 1∶1, 1∶2, 1∶4, and 1∶8). We found that blood CD4^+^ CD25^+^ Tregs isolated from secondary syphilis (84.0%±1.4%, P<0.05) and serofast syphilis (84.3%±3.0%, P<0.01) but not primary syphilis (74.5%±1.1%, P>0.05) and latent syphilis (73.8%±1.1%, P>0.05) patients exhibited significantly higher suppressive activity than healthy controls (66.3%±1.1%) at a 1∶1 (suppressor: responder) ratio (Figure 1C). Significant increases in suppressive effect of CD4^+^ CD25^+^ Tregs were also observed at ratios of 1∶2 and 1∶4 in secondary and serofast syphilis patients compared with healthy donors (Figure 1C). These data indicated that CD4^+^ CD25^+^ Tregs derived from secondary and serofast syphilis patients display enhanced suppressive function.

Since Transforming Growth Factor-β (TGF-β) is critical to Treg differentiation and suppressive function [37], [38], [39], we determined whether higher Treg frequency and function in syphilis patients were associated with serum TGF-β levels. It was shown that serum concentrations of TGF-β were significantly increased in patients with secondary (5.4±0.7 ng/ml, P<0.001) and, to a lesser extent, in primary syphilis patients (4.4±0.9 ng/ml, P<0.05), latent patients (4.6±0.5 ng/ml, P<0.01) and serofast patients (4.4±0.6 ng/ml, P<0.01) compared with healthy controls (1.1±0.2 ng/ml) (Figure 1D). There was a positive correlation between the percentage of circulating CD4^+^ CD25^high^ Tregs and serum TGF-β levels in these syphilis patients (r = 0.20, P<0.05, Figure 1E).

### Serum RPR Titers Are Positively Correlated to Treg Frequencies in Secondary, Latent and Serofast Syphilis

**Figure 2:**
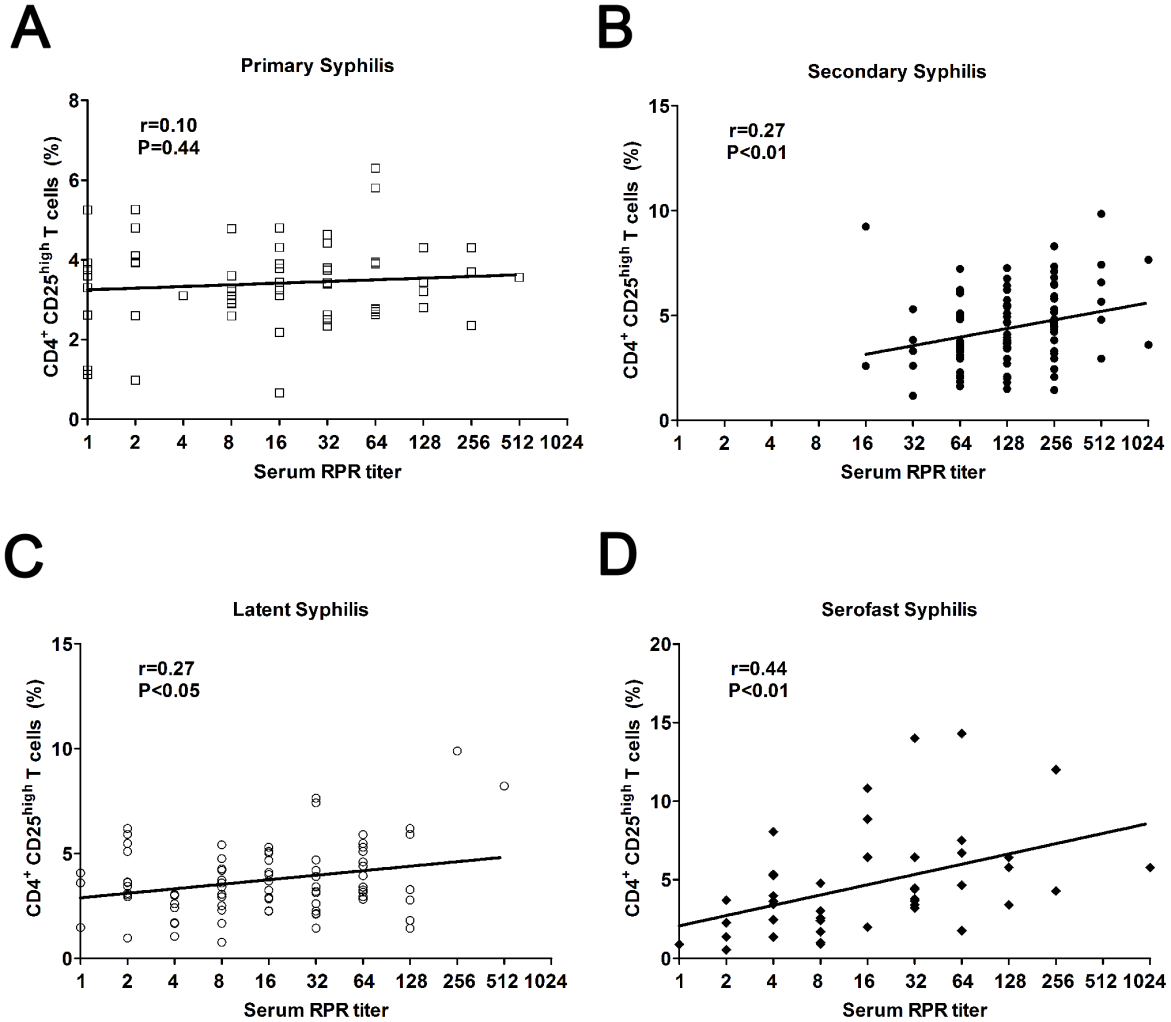
Association of circulating Treg numbers with serum RPR titers in primary, secondary, latent and serofast syphilis patients. Serum RPR titers are plotted against circulating CD4^+^ CD25^high^ Treg frequencies in primary (**A**, n = 64), secondary (**B**, n = 96), latent (**C**, n = 86) and serofast (**D**, n = 43) syphilis patients. Each dot represents an individual patient. The straight line in each graph is the result of linear regression analysis. Pearson's correlation coefficients (r) and P values are shown.

Nontreponemal test antibody titers usually correlate with disease activity [40]. We thus assessed whether serum RPR titers were associated with circulating Treg percentage in these syphilis patients. Pearson correlation analysis showed that there was a positive correlation between the percentage of circulating CD4^+^ CD25^high^ Tregs and serum RPR titer in secondary syphilis (r = 0.27, P<0.01, Figure 2B), latent syphilis (r = 0.27, P<0.05, Figure 2C) and serofast (r = 0.44, P<0.01, Figure 2D) syphilis patients, but no correlation in primary syphilis patients (r = 0.10, P = 0.44, Figure 2A).

### Circulating Treg Frequencies Are Higher in Neurosyphilis Patients than Syphilis Patients without Neurological Involvement

**Figure 3:**
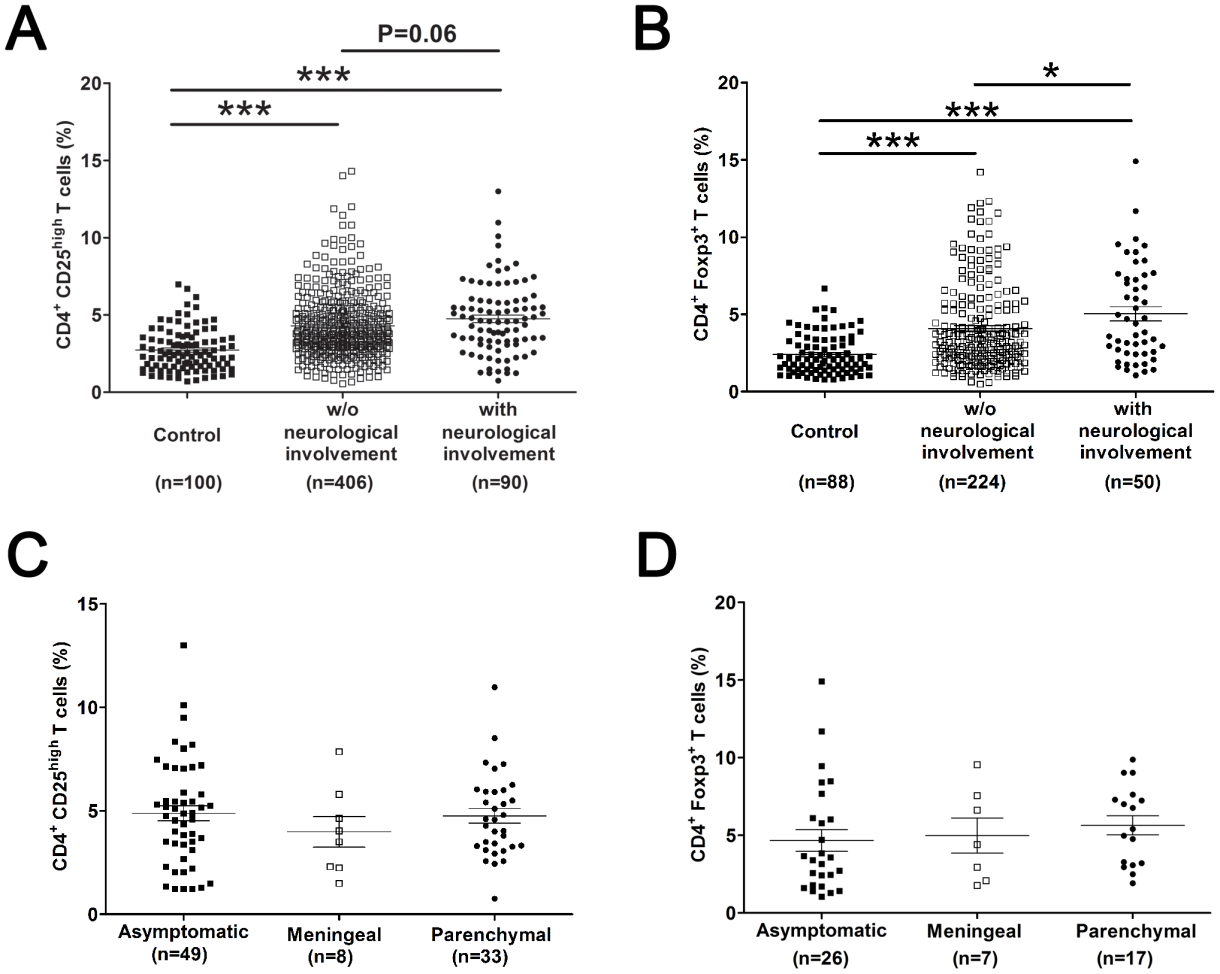
Treg frequencies in peripheral blood of neurosyphilis patients. The percentage of CD25^high^ (**A**) and Foxp3^+^ (**B**) among CD4^+^ T cells in peripheral blood of healthy controls, and syphilis patients without (including primary, secondary, latent and serofast syphilis) and with (including both asymptomatic and symptomatic neurosyphilis) neurological involvement are shown. The percentage of CD25^high^ (**C**) and Foxp3^+^ (**D**) among CD4^+^ T cells in peripheral blood of different types of neurosyphilis patients. Individual frequencies for every patient analyzed are shown. Results represent the mean ± SEM. A and B, Kruskal-Wallis test; C and D, One-way ANOVA. *, P<0.05, ***, P<0.001.

If untreated or treated improperly, some syphilis patients will progress to neurosyphilis. To investigate whether Tregs are associated with the progression of neurosyphilis, we analyzed Treg numbers in the peripheral blood of 49 asymptomatic and 41 symptomatic neurosyphilis patients. As shown in Figure 3A and 3B, syphilis patients with neurological involvement (including both asymptomatic and symptomatic syphilis patients) had higher percentage of CD4^+^ CD25^high^ Tregs (4.7%±0.2%, P<0.001) and CD4^+^ Foxp3^+^ Tregs (5.0%±0.4%, P<0.001) in peripheral blood compared with healthy individuals (2.7%±0.1% and 2.4%±0.1%, respectively). Compared to syphilis patients without neurological involvement (including primary, secondary, latent and serofast syphilis patients), there was a slight but not significant increase in CD4^+^ CD25^high^ Treg frequency in peripheral blood of neurosyphilis patients (P = 0.06) (Figure 3A), but the percentage of CD4^+^ Foxp3^+^ Treg were significantly higher (P<0.05) (Figure 3B). Among syphilis individuals with neurological involvement, there was no significant difference in CD4^+^ CD25^high^ Treg frequency (P>0.05) (Figure 3C) and CD4^+^ Foxp3^+^ Treg frequency (P>0.05) (Figure 3D) in peripheral blood among asymptomatic, meningeal, and parenchymal neurosyphilis patients.

### CD4^+^ T Cells Predominate in the Cerebrospinal Fluid of Neurosyphilis Patients

CSF mononuclear pleocytosis is one of diagnostic criteria for neurosyphilis [41], [42]. As expected, higher numbers of leukocytes were observed in asymptomatic (14±3 cells/µL), meningeal (35±15 cells/µL) and parenchymal (16±4 cells/µL) neurosyphilis patients compared to those from syphilis patients without neurological involvement (4±1 cells/µL) (P<0.001, P<0.001, and P<0.001, respectively) (Table 3). Among the CSF leukocytes, higher percentage of CD4^+^ T cells were found in patients with asymptomatic (41.8%±2.3%, P<0.01) and parenchymal (46.4%±2.1%, P<0.001) neurosyphilis compared with syphilis patients without neurological involvement (29.7%±2.4%) (Table 3). There was no significant difference in CSF CD4^+^ T cell frequency (P>0.05) among different types of neurosyphilis patients (Table 3).

**Table 3:** Absolute number of leukocytes, percentage and numbers of CD4^+^ T cells, Tregs, and TGF-β levels in CSF from different types of syphilis patients.

Type		Non-neurosyphilis	Asymptomatic	Meningeal	Parenchymal
**Leukocytes** a	**Cells/µL**	4±1	14±3	35±15	16±4
	***P*** b	—	<0.001	<0.001	<0.001
	***P*** c	—	—	<0.01	NS
	***P*** d	—	—	—	<0.01
**CD4^+^**	**%** e	29.7±2.4	41.8±2.3	49.7±2.6	46.4±2.1
	***P*** b	—	<0.01	NS	<0.001
	***P*** c	—	—	NS	NS
	***P*** d	—	—	—	NS
**CD4^+^**	**%** f	22.0±1.0	20.0±1.1	12.5±1.4	12.0±1.2
**CD25^high^**	***P*** b	—	NS	<0.05	<0.001
	***P*** c	—	—	<0.05	<0.001
	***P*** d	—	—	—	NS
**CD4^+^**	**Cells/µL**	0.1±0.1	1.2±0.2	0.9±0.3	0.5±0.1
**CD25^high^**	***P*** b	—	<0.001	<0.01	<0.01
	***P*** c	—	—	NS	<0.05
	***P*** d	—	—	—	NS
**TGF-β** g	**ng/ml**	8.2±1.7	10.7±2.0	3.4±0.9	2.8±0.5
	***P*** b	—	NS	<0.05	<0.05
	***P*** c	—	—	<0.05	<0.01
	***P*** d	**—**	**—**	**—**	NS

a CSF samples from 20 syphilis patients without neurological involvement, 23 asymptomatic, 7 meningeal and 14 parenchymal neurosyphilis patients were analyzed by flow cytometry.

b vs. syphilis patients without neurological involvement;

c vs. asymptomatic neurosyphilis patients;

d vs. meningeal neurosyphilis patients;

e percentage (%) among CD45^+^ leukocytes;

f percentage (%) among CD4^+^ T cells.

g TGF-β levels were examined in 41 syphilis patients without neurological involvement and 63 neurosyphilis patients.

**Abbreviations**: NS, Not Significant.

### CSF Treg Numbers Are Reduced in Symptomatic Neurosyphilis Patients

The average percentage of CD25^high^ Tregs in the CD4 compartment was 22.0%±1.0% for the patients without neurological involvement and did not differ from those with asymptomatic neurosyphilis (20.0%±1.1%, P>0.05). Both meningeal (12.5%±1.4%) and parenchymal (12.0%±1.2%) neurosyphilis patients showed pronounced decreases in CD4^+^CD25^high^ Treg percentage compared to syphilis patients without neurological involvement (P<0.05, P<0.001, respectively) and asymptomatic neurosyphilis patients (P<0.05, P<0.001, respectively) (Table 3). Due to preferential accumulation of CD4^+^ T cells in the CSF of neurosyphilis patients, both asymptomatic and symptomatic neurosyphilis patients have higher numbers of CD4^+^CD25^high^ Tregs than syphilis patients without neurological involvement. Interestingly, lower number of Tregs was observed in meningeal (0.9±0.3 cells/µL) and parenchymal (0.5±0.1 cells/µL) neurosyphilis patients than asymptomatic neurosyphilis patients (1.2±0.2 cells/µL). In addition, meningeal (3.4±0.9 ng/ml) and parenchymal (2.8±0.5 ng/ml) neurosyphilis patients had significantly lower CSF TGF-β levels than asymptomatic neurosyphilis (10.7±2.0 ng/ml) and syphilis patients without neurological involvement (8.2±1.7 ng/ml), indicating that decreased CD4^+^ CD25^high^ Treg frequencies in CSF of symptomatic neurosyphilis patients may be associated with low CSF TGF-β concentration.

## Discussion

Syphilis is a multistage chronic disease, which can cause damage to diverse tissues and organs. An influx of immune cells to skin lesions of early syphilis patients not only mediates bacterial clearance but also lead to tissue damage and clinical symptoms [9], [10], [43], [44]. Our prior study has shown that immune cells can also infiltrate into the CSF of syphilis patients [45]. However, this study was limited because of a small number of patients (n = 32), selected patient populations (latent syphilis and neurosyphilis) and lack of characterization of neurosyphilis patients [45]. In the present study, a total of 431 syphilis patients without neurological involvement (including 20 latent syphilis patients in the previous report) and 100 neurosyphilis patients (including 12 patients in the previous report) were included. This larger number of syphilis patients enables further stratification according to stage and symptoms.

Interestingly, we observed an accumulation of CD4^+^ T cells in the CSF of both asymptomatic and symptomatic neurosyphilis patients, which were consistent with several previous reports showing that CD4^+^ T cells were the primary responders to *T. pallidum* in syphilis lesions [8], [9], [46]. CD4^+^ T cells can be divided into a variety of effector subsets, including classical Th1 cells and Th2 cells, the more recently defined Th17 cells, follicular helper T cells, and regulatory T cells [47]. Though we did not elucidate the precise identity of the CD4^+^ T cell subset, we observed a decreased frequency of CD4^+^ CD25^high^ Tregs in the CSF of symptomatic neurosyphilis patients compared with those of non-neurosyphilis and asymptomatic neurosyphilis patients. Given the important role of Tregs in controlling immune-mediated tissue damage, our results suggest that the CNS damage in neurosyphilis patients may be due to an uncontrolled host immune response. A local decrease in Tregs may facilitate CNS injury in neurosyphilis patients. A similar scenario has been observed in other CNS disorders [48], [49].

*T. pallidum* can establish persistent infection by promoting Treg response in early stage of syphilis. In marked contrast to reduced local Treg response in symptomatic neurosyphilis, we found that Treg numbers in circulation of neurosyphilis patients were even higher than early syphilis patients without neurological involvement. This finding suggests that suppression of the systemic immune response against *T. pallidum* may favor neurological progression. Consistent with this notion, studies have found that HIV-positive people infected with *T. pallidum* are more likely to develop neurosyphilis, even during the early stages of infection [5], [50].

The mechanisms underlying Treg differences among syphilis patients are poorly understood. Given that TGF-β was implicated in modulating Treg differentiation and activity [37], [38]; we investigated whether the frequency and functional status of Tregs were associated with this cytokine. We confirmed that serum from the patients with secondary and serofast syphilis did express significantly higher levels of TGF-β than those of healthy control subjects, which may be related to the increased frequency and enhanced function of Tregs in these patients. Lower TGF-β levels were observed in CSF of symptomatic neurosyphilis patients than asymptomatic neurosyphilis patients, which may be associated with a decrease in CSF Treg numbers.

**Figure 4:**
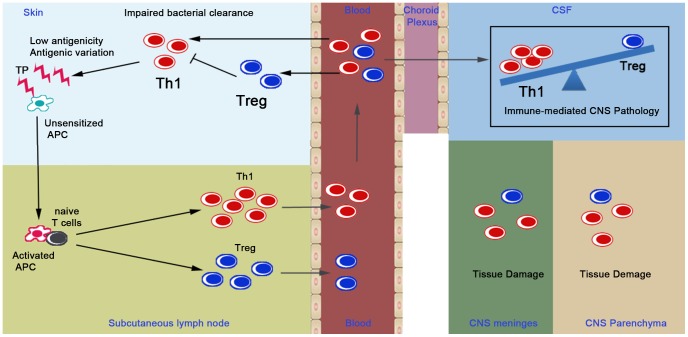
Schematic diagram summarizing the role of Treg in syphilis. APC, antigen presenting cells; Th1, T helper cell type 1; Treg, regulatory T cells; CSF, cerebrospinal fluid; CNS, central nervous system; DC, dendritic cells.

We propose a model to summarize the role of T cell subsets in the pathogenesis of syphilis in Figure 4. *T. pallidum* penetrates through abraded skin where antigen presenting cells (APC), such as dendritic cells (DC), process the bacteria and then migrate to the subcutaneous lymph nodes. These activated APC [51] may present *T. pallidum*-derived antigens to naïve T cells and induce production of Th1 [9] and Treg [18], which enter the peripheral blood and circulate widely throughout the body. *T. pallidum* has the ability to preferentially enhance the generation of Tregs through TGF-β [18], which may impair Th1 function to favor bacterial persistence in the circulation and skin. Antigenic variation and poor antigenicity also enable *T. pallidum* to evade cell mediated immune response [13], [15], [16]. However, a defective accumulation of Tregs in the CNS (Table 3) may fail to suppress T cell-mediated inflammation and tissue damage in the meninges and parenchyma of brain and spinal cord, resulting in neurological symptoms and signs.

In our study cohort, there are differences in sex distribution among syphilis patients of different stages: 78.0% neurosyphilis patients (78/100) were male, while only 35.1% serofast syphilis patients (34/97) were male. However, there was no significant difference in blood and CSF CD4^+^ CD25^high^ Tregs between males and females in each group (data not shown), which indicating that the Treg differences between stages were not due to gender preference. Serofast status represents a clinical challenge for treatment of syphilis. There is no universally accepted definition of “serofast”. The definition of “serofast” in this manuscript is “having had an appropriate 4-fold titer decline after treatment, but not reverting to seronegative”. Although these syphilis patients meet criteria for being adequately treated, we and others have shown that such “serofast” patients can progress to neurosyphilis [52], [53], suggesting that they still harbor *T. pallidum*. The immune status of serofast patients is unclear. A recent study reported that HIV-infected patients are at increased risk for serofast state after treatment [54]. Our results showed that these patients had enhanced circulating Treg numbers and suppressive function, also suggesting serofast status may be associated with a systemic immune suppression.

There are several limitations in the analysis of Treg activity in this study. First, future studies should examine Foxp3 expression and define the functional status of CD4^+^ CD25^high^ Tregs in CSF in neurosyphilis patients. We were not able to conduct such studies because of the limited availability of CSF T cells. In addition, studies of Treg loss-of-function and gain-of-function are needed to further explore their role in syphilis, but these experiments have been hampered by inherent difficulty in conducting immunologic studies of syphilis in experimental animal models [9].

In conclusion, our findings demonstrate for the first time that neurological progression in syphilis patients is associated with increased circulating Tregs and CSF CD4^+^ T cells and reduced local Treg response is implicated in the development of symptoms in neurosyphilis patients.
